# An insight into the draft genome of the Oriental rat flea, *Xenopsylla cheopis*, together with its *Wolbachia* endosymbiont

**DOI:** 10.1186/s12864-025-11759-8

**Published:** 2025-07-01

**Authors:** Stephen Lu, David M. Bland, Eric Dahlstrom, Neelam Redekar, Melina G. Guizzo, Kent Barbian, B. Joseph Hinnebusch, José M. C. Ribeiro

**Affiliations:** 1 Section of Vector Biology, Laboratory of Malaria and Vector Research, National Institute of Allergy and Infectious Diseases, National Institutes of Health, 12735 Twinbrook Parkway, Room 2E-28, Twinbrook III Building, MSC 8132, Bethesda, MD 20892-8132 USA; 2 Laboratory of Bacteriology, Rocky Mountain Laboratories, National Institute of Allergy and Infectious Diseases, National Institutes of Health, Hamilton, MT 59840 USA; 3 Genomics Unit, Research Technologies Branch, Rocky Mountain Laboratories, National Institute of Allergy and Infectious Diseases, National Institutes of Health, Hamilton, MT 59840 USA; 4 Integrated Data Sciences Section, Research Technologies Branch, National Institute of Allergy and Infectious Diseases, National Institutes of Health, Bethesda, MD 20892 USA

**Keywords:** *Xenopsylla cheopis*, *Wolbachia*, Genome, Symbiont, Flea, Plague

## Abstract

**Background:**

The Oriental rat flea, *Xenopsylla cheopis*, is a main vector of plague caused by the bacterium *Yersinia pestis.* Transcriptomic analysis of this insect and the interaction between *Yersinia* and the flea digestive tract have been the subject of several studies. However, to develop more refined studies on this vector in the future, we sequence and describe a draft genome of the rat flea *Xenopsylla cheopis*, discuss the physiological implications of its genetic features, and compare them with the only other sequenced member of the Siphonaptera, the cat flea, *Ctenocephalides felis.*

**Results:**

Sequencing data from both long and short reads were assembled into 7,694 contigs, from which 95,638 putative coding sequences (CDSs) were extracted and functionally annotated, providing insights into various aspects of flea physiology. This includes the identification of putative salivary proteins, such as acid phosphatases and FS-H/I, associated with blood acquisition; classification of multiple serine peptidases likely representing the primary digestive enzymes of *X.cheopis*; and the identification of all enzymes involved in heme biosynthesis, as well as heme oxygenases and unique heme-binding proteins potentially involved in heme detoxification. Comparison of detoxification-related genes—namely those in the cytochrome P450, carboxylesterase, and glutathione S-transferase families—with homologs from the cat flea (*C. felis*) revealed the presence of a platelet-activating factor (PAF) acetyl hydrolase that appears to be unique to rat fleas, cat fleas, and human head and body lice, but is absent in other blood-feeding arthropods. Additionally, we identified key components of immune-related pathways known from other arthropods, including the Toll, IMD, and JAK/STAT pathways. Finally, a contig encoding a novel bacterium was discovered within the assembled flea genome. Phylogenetic analysis of the *Wolbachia* endosymbiont in *X. cheopis* suggests it is closely related to the *Wolbachia* strain found in *Drosophila melanogaster*.

**Conclusions:**

The disclosure of the *X. cheopis* genome, together with its *Wolbachia* symbiont, should advance research on the biology of this vector.

**Supplementary Information:**

The online version contains supplementary material available at 10.1186/s12864-025-11759-8.

## Background

Fleas are a morphologically unique, highly specialized, monophyletic group of wingless insects. The roughly 2,500 species (_~_16 families, 246 genera) constitute the Siphonaptera, a relatively recent insect order that emerged in the Cretaceous period that is most closely related to the Mecoptera (scorpion flies) and Diptera [1–3]. Adult fleas, both males and females, are obligate blood-feeding ectoparasites of mammals (~ 75% of species) and birds present in a wide range of ecological habitats on all continents except Antarctica. The great diversity of flea species correlates with mammalian diversification during their shared evolutionary history [1].

Flea taxonomy, based on morphological characteristics, has been well studied but despite the medical, veterinary, and economic importance of fleas, their genomics and phylogenetics have received relatively little attention compared to other insect orders. The first genome sequence of a flea species, that of the cat flea *Ctenocephalides felis* (nine chromosome pairs, ~ 650 Mb), was reported in 2020, and to date is the only publicly available complete annotated flea genome. Striking findings of this study were the extraordinary amount of gene duplication present in the cat flea genome as well as megabase-length differences in genome size among individual fleas of the same species [4].

Here we report a scaffold-level assembly and annotation of the genome of *Xenopsylla cheopis*, a rat flea widely present in warmer regions throughout the world which is of particular importance because it is the major vector of *Yersinia pestis*, the bacterial agent of bubonic plague to humans. It is also the principal vector of *Rickettsia typhi*, the cause of murine typhus in humans. Previous studies showed that *X. cheopis* has a chromosome complement of 2n = 18, X_1_ × _1_ × _2_ × _2_ (female) and 2n = 17, X_1_ × _2_Y (males) but with extensive variation in genome size between individuals. A total of 95,638 coding sequences (CDS) were detected, analyzed, and annotated. Proteins predicted to be central to blood meal digestion, detoxification, immune system pathways, and salivary gland output and function were investigated further. The large number of transposable elements (TEs) present in the genome were characterized. A *Wolbachia* endosymbiont genome was also found. Future comparative analyses may provide insight into intrinsic factors that account for the very high vector competence of *X. cheopis* for *Y. pestis* transmission relative to the very low vector competence of *C. felis* [5].

## Methods

### Flea samples

*Xenopsylla cheopis* colonies were derived from fleas collected in Baltimore, MD and Los Angeles, CA and have been maintained at the Rocky Mountain Laboratories for roughly 30 years. A single, newly emerged female, and a single male were isolated and allowed to mate. F1 adult female progeny of these two parents were starved for four days and stored at −80° C prior to DNA extraction.

### DNA extraction

High molecular weight total genomic DNA was extracted from single fleas using a MagAttract HMW kit (Qiagen, Germantown, MD) following previously described methods [6] with few modifications. Briefly, individual fleas were triturated for 30 s in a 187 µl mixture containing 100 µl PBS; and 10 µl Proteinase K, 2 µl RNase A, and 75 µl Buffer AL (kit components) using the textured mortar/pestle PowerMasher II (DiagnoCine, Hackensack, NJ). The triturates were incubated for 2 h at 25 °C with gentle mixing every 15 min. Following subsequent bead-binding and wash steps, the gDNA was eluted twice with 65 µl and 35 µl of Buffer AE, each at 25^°^ C at 1400 rpm for 8 min. The elutions were combined and stored at 4^°^ C. Total gDNA yield was assessed with a Qubit fluorometer using the dsDNA HS assay kit (Thermo Fisher Scientific, Waltham, MA) and with a Fragment Analyzer (Advanced Analytical, Ankeny, IA) using the DNF-464-22 HS Large Fragment kit. Size distribution was viewed using ProSize software 2.0 (Advanced Analytical). Total gDNA from a single flea sample with the largest yield (472 ng), largest size distribution (average 23,868 bp), and least amount of shearing was subsequently used for library preparation using the SMARTbell Express Prep kit v2.0 (Pacific Biosciences, Menlo Park, CA).

### Genome sequencing

Whole-genome sequencing of total DNA from this single flea was performed using the PacBio Sequel system (Pacific Biosciences) following methods previously described [6]. The HMW gDNA was not sheared, the DNA damage repair time was extended by 1.5 h, ligation was performed overnight (16 h), a nuclease treatment was not performed, and a 0.45X AMPpure XP (Beckman Coulter, Brea, CA) cleanup after ligation was performed in addition to a size selection with diluted (40% v/v) AmPure XP beads to remove < 3 kb SMRTbell templates. Final library size (average 14,333 bp) and concentrations were assessed on the Fragment Analyzer (Advanced Analytical) and Qubit 3.0 fluorometer before proceeding with primer v4 and polymerase 3.0 binding following the manufacturers recommendations. A total of seven SMRTcells were run following on-plate diffusion loading with concentrations ranging from 7 to 10 pM using the Sequel Sequencing kit 3.0, 10-hour movie times, CLR sequencing mode, 30 min pre-extension, and SMRTlink v6.0 software (Pacific Biosciences).

### Genome assembly

The PacBio subread BAM files were first converted to FASTQ (Samtools v1.9) and then to FASTA format (SeqKit fq2fa, v0.12.1). FASTA sequences were used to generate flea assembly with the Raven (v1.1.5) assembler in graphical fragment assembly format. Initial genome completeness was estimated against endopterygota_odb10 lineage dataset with BUSCO (v4.1.3, genome mode) [7]. Prior to assembly polishing, the PacBio subreads were blasted (blastn, v2.81) against known flea mitochondria and those reads with an e-value of 0.001 or greater, along with alignment lengths of 75% or greater were removed. Additionally, the reads were run through removesmartbell.sh (BBMAP v38.96) [8] to check for and remove any end adapters from identified reads. Next, two rounds of polishing were run on the PacBio assembly using Minimap2 (v2.14-r883) [9] to align the PacBio subreads back to the assembly, then Racon (v1.4.3) [10] to generate a higher quality genomic consensus. This consensus sequence was then checked and corrected for both gaps and INDELS using Pilon (v1.22) [11].

DNA extracted and prepared as described above from a second individual F1 (sibling) female flea was sequenced using the Illumina NextSeq 550 system (Illumina, San Diego, CA). Library preparation with 200 ng HMW gDNA from this flea was performed using a TruSeq Nano DNA kit (Guide#15041119-REV.D) following the manufacturers recommendations. The gDNA sample was sheared to a target insert size of 550 bp, and the final library was visualized using a BioAnalyzer HS chip (Agilent Technologies) and quantified using the KAPA Library Quant Kit - Illumina Universal qPCR Mix (Kapa Biosystems, Wilmington, MA) and the CFX96 Real-Time System (BioRad, Hercules, CA). The library was diluted to 2 nM stock and sequenced on an Illumina NextSeq 550 instrument as paired-end 2 × 150 bp reads. The Illumina reads were trimmed and filtered for adapter sequences using Cutadapt (v1.12) [12], then passed through fastq_quality_trimmer and fastq_quality_filter (FastX toolkit, v0.0.14) [13] to remove both low quality and too-short reads. Next, blastn, as above, was used to identify and remove mitochondrial reads. A final round of error correction was then run using the Illumina short reads. The Illumina reads were first mapped to the polished PacBio assembly contigs using BWA-MEM (BWA, v0.7.17-r1188) [14]. Pilon was then run again using the Illumina-mapped reads output to generate the final assembly [11].

### Genome annotation

#### Flea genome

The BRAKER/Augustus pipeline [15] was used to obtain the putative coding sequences (CDS) from the *X. cheopis* genome. The program was trained to find the CDS using a subset of the predicted proteins available from the Transcriptome Shotgun Assembly (TSA) database from the National Center for Biotechnology Information (NCBI) (Bioprojects PRJNA601490 and PRJNA760518) totaling 8,415 sequences containing a starting methionine and stop codon. This approach resulted in the discovery of 107,197 coding sequences (CDS). After removing duplicated sequences, we obtained a final set of 95,638 putative CDS. The BUSCO program (version 5.0.0) [7] was run with the BRAKER predicted protein sequences against the lineage dataset insecta_odb10, created on 2020-09-10, from 75 species and 1,367 BUSCOs. Quast [16] was used to assess the quality of the genome assembly. The CDS were also compared to the RepBase [17] and UniprotKB protein [18] databases to identify and classify TEs. To classify genes according to their functional class, the deducted protein sequences were compared using blastp to a subset of the GenBank database containing sequences from the Diptera, to the UniprotKB [18] database, to the Expasy Enzyme (EC) [19] database and to the MEROPS [20] database. Rpsblast was used to search the protein sequences against conserved motifs from the PFAM [21], SMART [22], KOG [23] and CDD [24] databases. Fasta files from the ESTHER database [25] were used to classify the carboxylesterases, when blastp matches resulted in a minimum of 90% coverage and a minimum of 35% identity.

#### Transposable elements

Transposable elements were identified by the programs Repeat Masker [26] and RepeatModeler2 version 2.01 [27]. For comparison, we have also identified TEs in the genome of the cat flea *Ctenocephalides felis* (NCBI accession RefSeq GCF_003426905.1). Additionally, genes identified by the Braker-Augustus program in the genome of *X. cheopis* were compared by blastp to the protein sequences found in the Repbase database [17].

#### Symbiont genome

Contig_730 was identified to derive from a bacterial organism following its matches to peptides from Rickettsiales found in the RefSeq [28] protein database from the NCBI. The NCBI Prokaryotic Genome Annotation Pipeline (PGAP) [29] was used to obtain its genome annotation. The phylogenetic analysis was done using the GToTree phylogenomic method [30]. The coding sequences (mRNA) of three *Anaplasma* and 186 *Wolbachia* bacteria were downloaded from the NCBI Refseq database (Supplementary File 2) and submitted to the program GToTree. The sequences for each genome were compared by HMMER [31] to 117 single copy target gene hidden Markov models (hmm) of alpha-protobacteria. Of the 190 genomes analyzed, an average of 96 SCG were used per genome in the final alignment, with a maximum of 100 and a minimum of 62. There were 96 SCG found on the *X. cheopis* symbiont genome. The concatenated coding sequences were aligned by Muscle [32], and the tree was constructed by FASTTREE [33]. The tree rooted on the *Anaplasma* branch was drawn with Mega X [34]. For the identification of vitamin B biosynthetic pathways, we compared the *X. cheopis* symbiont coding sequences to the COG [35] and UniprotKB [18] databases. We also compared, using blastp, the predicted proteins of the *Wolbachia* endosymbiont of *Cimex lectularius*, which has complete biosynthetic pathways to most members of the vitamin B family [36], to the predicted proteins of the *X. cheopis* symbiont.

## Results

The assembled *Xenopsylla cheopis* genome (excluding the bacterial genome contig discussed below) contains 7,694 contigs totaling 691,786,654 bases, with N50 = 214,872 bases and L50 = 836 bases. The BUSCO analysis [7] revealed 92.8% complete BUSCOs, 83.4% of which are complete single copy and 9.4% are complete and duplicated. 3% were fragmented and 4.2% were missing.

Gene prediction using the BRAKER/Augustus pipeline identified 95,638 putative coding sequences (CDS). Further annotation of the 95,638 predicted CDS enabled their classification into 24 functional groups. The most prevalent category was “unknown,” comprising 45,270 CDS (47.33%). These include sequences similar to previously deposited genes of unknown function, as well as sequences with no significant similarity to known genes—suggesting the presence of potentially novel proteins.

To identify putative orthologs conserved between *X. cheopis* and *C. felis*, we performed a reciprocal smaller distance (RSD) [37] analysis. This analysis identified 8,805 conserved CDS between the two flea species (Supplementary file 3). Functional classification of these orthologs revealed that the majority (42.61%) also fall into the “unknown” category, followed by sequences predicted to encode “secreted” proteins (8.78%). The substantial proportion of “unknown” CDS in both flea genomes underscores the significant gaps in our current understanding of flea protein function and highlights a vast reservoir of uncharacterized genetic diversity.

In addition to the *X. cheopis* coding sequences (CDSs), we identified a contig representing a potential endosymbiont belonging to the *Wolbachia* genus. In the following sections, we present the genome of this bacterial organism, followed by an analysis of transposable elements within the *X. cheopis* genome. We also examine genes associated with specific physiological functions of *X. cheopis*, including those involved in blood acquisition and processing, heme management, detoxification, and immune responses.

### The *X. cheopis endosymbiont*

To identify possible contigs deriving from bacterial sources rather than from the *X. cheopis* genome, we determined the open reading frames (ORFs) from all the 7,721 contigs and compared them by blastp to bacterial protein and Insecta databases. Contig_730 had 1,503,042 bases in length and best matched proteins from the *Rickettsiales* class. The PGAP pipeline identified 1,382 CDSs on Contig_730, 1,224 of which coded for proteins, 35 genes coded for tRNAs, 3 coded for rRNAs, 1 represented a ncRNA and 157 coded for pseudogenes. BUSCO analysis [7] against the lineage dataset rickettsiales_odb10 indicated 97% complete BUSCOs, 94.5% complete and single-copy, and 2.5% complete and duplicated BUSCOs.

To determine the taxonomic status of this bacterium, we used the GToTree [30] phylogenomics approach. The resulting phylogenetic tree (Supplementary File 1, Supplementary Figure 1) displayed branches with strong bootstrap support for supergroups A, B, C, D and F [38–40]. The endosymbiont of *X. cheopis*, and the *Wolbachia* endosymbionts of the cat flea *Ctenocephalides felis*, *Folsomia candida*,* Pentalonia nigronervosa* and *Howardula sp*. appear as individual branches. The symbiont of *F. candida* was proposed to belong to a new supergroup E [39, 41], while the symbiont for *Pentalonia* was attributed to supergroup M [42]. Two strains of the *C. felis Wolbachia* symbiont, analyzed by 16 S rRNA, were attributed to supergroups V and W [38]. The *Howardula* [43] as well as the *X. cheopis* symbiont did not cluster with any supergroup.

### *Wolbachia* biosynthetic capability

*Wolbachia* are obligate intracellular bacteria harbored by a significant portion of arthropod species and filarial nematodes worldwide [44]. Primarily vertically transmitted, these endosymbionts play complex/varied roles in their hosts biology, with a wide range of interactions, from parasitic to mutualistic [45, 46]. While several *Wolbachia* strains manipulate the host reproduction to spread vertically, other strains which do not have this capacity are equally transmitted efficiently [47]. The genome of the *Wolbachia* endosymbiont of *X. cheopis* revealed genes for the biosynthetic pathway of six vitamins of the B complex: biotin, riboflavin, folic acid, pyridoxine, thiamine and nicotinic acid (Table 1). The riboflavin (B2) pathway was the only one containing all the genes for its production. For folic acid (B9), the final gene of the pathway was missing. In contrast, only the final enzyme for the thiamine (B1) biosynthesis has been retained. Similarly, only the final two genes of the pathway for pyridoxine (B6) were present. The biotin (B7) biosynthesis pathway as well as the nicotinic acid (B3) were also incomplete. Genes for the biosynthesis of pantothenic acid (B5) were absent. In the context of cofactor biosynthesis, genes for FAD, NADP + and CoA were also present in the endosymbiont genome (Table 2), but only the pathway for FAD production has been fully retained.

**Table 1:** Biosynthetic pathways for B vitamins in *Wolbachia* endosymbiont of Xenopsylla Cheopis

Vitamin	Gene	Status	Transcript ID
Biotin (B7)	bioC	Present	WOB49_000199
	bioH	Absent	
	bioF	Present	WOB49_000198
	bioA	Present	WOB49_000855
	bioD	Present	WOB49_000200
	bioB	Present	WOB49_000197
Thiamine (B1)	thiC	Absent	
	thiD	Absent	
	thiE	Absent	
	thiL	Present	WOB49_001054
Riboflavin (B2)	ribA	Present	WOB49_001111
	ribB	Present	WOB49_001186
	ribC	Present	WOB49_001360
	ribD	Present	WOB49_000135
	ribE	Present	WOB49_001360
	ribH	Present	WOB49_001355
Folic acid (B9)	folE	Present	WOB49_000108
	folB	Present	WOB49_001089
	folK	Present	WOB49_000577
	folC	Present	WOB49_000252
	folD	Present	WOB49_001263
	folA	Absent	
Pyridoxine (B6)	epd	Absent	
	pdxB	Absent	
	serC	Absent	
	pdxA	Absent	
	pdxJ	Present	WOB49_000551
	pdxH	Present	WOB49_000139
Pantothenic acid (B5)	ilvE	Absent	
	panB	Absent	
	panE	Absent	
	panC	Absent	
	panD	Absent	
Nicotinic acid (B3)	nadB	Present	WOB49_000645
	nadA	Absent	
	nadC	Absent	
	pncB	Absent	

**Table 2:** Biosynthetic pathways for cofactors in *Wolbachia* endosymbiont of *Xenopsylla cheopis*

**Cofactor**	**Gene**	**Status**	**Transcript ID**
FAD	ribF	Present	WOB49_000846
NADP+	pncB	Absent	
	nadM	Absent	
	nadE	Present	WOB49_000242
	ppnK	Absent	
CoA	coaA	Absent	
	coaBC	Absent	
	coaD	Present	WOB49_001315
	coaE	Present	WOB49_000602

### *Xenopsylla cheopis* transposable elements

Transposable elements (TEs) are virus-like sequences consisting of a single DNA sequence coding for 1 or more genes that infect the genomes of most organisms. Class I elements, or retrotransposons, code for several genes, always including a reverse transcriptase which is a key component of their replication mechanism. Class II elements, or DNA-transposons, replicate by a cut and paste mechanism that does not include an RNA intermediate. They have inverted terminal repeats that are recognized by the product of their singly expressed gene coding for a transposase. These two classes are further subdivided into several families [reviewed in [48]. Class I elements are subdivided into two main subclasses, the Retroelement and the LTRs (Long Terminal Repeats) subclasses. The Repeat Masker program revealed that the flea *X. cheopis* genome has 22% of its content represented by TEs, 14% of the genome coverage (GC) being derived from class I elements and 8% from class II elements. Among the class I elements, Retroelements occupy 13% of the genome, of which the Line and RTE families contribute to 9.4% of the GC. LTRs contribute to 1% of the GC, with the most abundant families deriving from the Gypsy and Bel/Pao families which together account for 9.95% GC. The most abundant Class II elements are represented by the TC1-Pogo and P-element families (Supplementary File 4).

### Salivary proteins of the *X. cheopis genome*

In the current genome assembly, we identified several putative secreted proteins that have been previously associated with blood acquisition in the saliva or salivary glands of other blood-feeding arthropods. Among these proteins are those bearing similarities to enzymes such as apyrases, antigen-5, peptidases inhibitors, acid phosphatases, lipases, and members of the unique FS-H/I antigen family.

Previous transcriptomic and proteomic studies have shown that acid phosphatases and members of the FS-H/I family are among the most abundant transcripts and proteins within the flea salivary gland [49, 50]. Notably, of the 10 acid phosphatases identified, six were found in tandem on the same contig (4606). Similarly, the 22 putative FS-H/I members were clustered within two contigs (1970 and 3604). When compared to the *C. felis* genome, we identified 767 putative secreted proteins conserved between both fleas, including putative peptidases and peptidases inhibitors, lipases and acid phosphatases.

### Genes related to peritrophic matrix formation and blood digestion

In many blood-feeding insects, the blood meal is enclosed in the midgut lumen by a semi-permeable acellular membrane composed of chitin, glycosaminoglycans and proteins. This specialized membrane, known as the peritrophic membrane or peritrophic matrix (PM) [51], allows the passage of solutes up to 30 kDa [52] such as serine peptidases like trypsin and chymotrypsin, to gain entry to the intraperitrophic space and initiate protein degradation [53]. Beyond its role in containing the meal bolus and regulating the digestive process [54], the PM can also serve as a barrier, providing protection to the insect midgut. Adult *X. cheopis* and *C. felis* do not produce a PM in response to blood ingestion, despite producing a number of proteins with chitin-binding domains, known as peritrophins, that are integral to PM formation in other arthropods [55–57]. There is some variability in the tissue specificity of the peritrophin transcripts, such as in the Malpighian tubules and the hindgut, but many are produced in the midgut epithelium [57]. Overall, we identified 14 annotated peritrophins in the *X. cheopis* genome, transcripts from 7 of these were expressed in the midgut and/or the proventriculus within 4 h of a blood meal (Supplementary File 5) [56]. All the rat flea peritrophins contain at least one PFAM CBM14 peritrophin A chitin-binding domain, consisting of a 6-residue cysteine motif [58]. We also identified a mucin-like protein in the *X. cheopis* genome, expressed in the midgut [56], that contained a CBM 14 peritrophin domain and 10 putative O-linked glycosylation sites (Supplementary File 5). Mucin-like proteins are characterized by an increased abundance of serine and threonine residues, important for O-linked glycosylation, and members of this family are incorporated in the PM in other arthropods (Supplementary File 5) [59]. The rat flea mucin-like protein was similar to the MPL-1 mucin-like protein identified in *C. felis* [57]. However, *C. felis* MPL-1 is exclusively expressed in the hindgut and/or Malpighian tubule tissue.

We identified several serine peptidases in the rat flea genome, including 37 trypsin-like, and 19 chymotrypsin-like serine peptidases, alongside two aminopeptidases and three carboxypeptidases (Supplementary File 6, Supplementary spread­sheet 1). Prior investigations exploring the flea midgut transcriptome before and after a blood meal revealed upregulation of several serine peptidases in both *C. felis* [60] and *X. cheopis* [56]. Together, these findings corroborate the notion that fleas, akin to mosquitoes and other blood-feeding insects, rely on serine peptidases as their main digestive enzymes. However, in the absence of a peritrophic membrane, the kinetics of blood meal digestion in fleas are likely to differ from those observed in mosquitoes. Future research characterizing the molecular biology and transcriptional regulation of rat flea digestive peptidases will yield more detailed insights into flea digestion physiology.

### Genes related to heme management

Lysis of red blood cells and subsequent enzymatic processing of hemoglobin by fleas likely results in the release of considerable amounts of heme. Heme and the iron contained within are important nutrients and signaling molecules in metabolism and oogenesis but can also generate cytotoxic reactive oxygen species (ROS) [61]. Because *X. cheopis* ingest blood, lyse red blood cells (RBCs), and digest the blood meal rapidly, management of dietary heme is likely essential for rat fleas [55].

Like most blood feeding arthropods, with the exception of the hard ticks, the rat flea genome contains all 8 genes encoding enzymes in the heme biosynthesis pathway as well as heme oxygenase important for catabolism (Table 3) [62]. The presence of these genes indicate that rat fleas can synthesize their own heme B and catalyze its oxidative degradation into biliverdin, carbon monoxide, and ferrous iron (Fe^2+^) for use in various physiologic processes. Aggregation and sequestration of heme is a common strategy among blood-feeding arthropods to limit its cytotoxic effects. We identified a putative heme-binding protein in the rat flea genome that does not share significant similarity with any of those from other blood-feeding arthropods. The putative *X. cheopis* heme-binding protein contains a beta-barrel heme-binding domain with similarity to the C-terminal region of human THAP4 (cTHAP4) [63]. It has been hypothesized that cTHAP4 binds nitric oxide (NO) and allows THAP4 to function as an NO-responsive transcriptional regulator. Furthermore, cTHAP4 has similar binding properties to nitrobindin, which has been proposed as a ubiquitous family of 10-stranded β-barrel heme-binding proteins [64, 65]. Interestingly, this putative heme-binding protein was not detected in the genome of the cat flea (Table 3). Cat fleas feed frequently, turning over the contents of their midgut and defecating partially processed blood many times per day. In contrast, the rat flea only feeds a few times per week, taking a comparatively longer time to digest their blood meal [66]. The longer retention time of heme in the midgut of the rat flea may necessitate use of additional strategies to mitigate the effects of heme, such as expression of a heme-binding protein. Further experimental work is necessary to validate this protein’s heme-binding activity and determine if it moderates ROS production in the flea midgut.

**Table 3:** Genes involved in *X. cheopis* heme management

Contig Name	Encoded Protein	Homolog present in the cat flea genome	Detected in Midgut Transcriptome (≥ 5 FPKM AVG)^*^	E Value	Coverage (%)
Heme Biosynthesis					
g36078.t1	5-aminolevulinate synthase, erythroid-specific, mitochondrial isoform X1 [ALAS]	Yes	NA	0	87
g35454.t1	delta-aminolevulinic acid dehydratase [PBGS]	Yes	Yes	6.00E-163	96
g30288.t1	uncharacterized protein LOC5573216 [HMBS]	Yes	NA	2.00E-52	85
g96981.t1	uroporphyrinogen-III synthase [UROS]	Yes	Yes	3.00E-46	90
g77830.t1	uroporphyrinogen decarboxylase isoform X1 [UROD]	Yes	Yes	7.00E-169	100
g9798.t1	oxygen-dependent coproporphyrinogen-III oxidase [CPOX]	Yes	Yes	4.00E-176	96
g47668.t1	protoporphyrinogen oxidase [PPOX]	Yes	Yes (below 5)	3.00E-150	85
g80088.t1	ferrochelatase, mitochondrial [FECH]	Yes	Yes	0	94
Heme Catabolism					
g57516.t1	Heme oxygenase 1 partial [HO]	Yes	No	5.00E-60	75
g43173.t1	Heme oxygenase 1 partial [HO]	Yes	Yes	1.00E-70	90
g104194.t1	Heme oxygenase 1 partial [HO]	Yes	Yes	9.00E-71	90
Putative Heme Binding/Extracellular Antioxidant					
g49535.t1	Putative heme-binding protein, [cTHAP4-like]	No	Yes	4.00E-54	97
g13160.t1	Mucin-like protein 1	Yes	Yes	0	58

^*^Determined from midgut transcriptome data in reference [56]

### Immunity-related genes

Insects have highly developed systems to sense and inactivate foreign microbes; over 400 genes of *Drosophila melanogaster* have been categorized as having known or potential roles in immunity [67]. Infection elicits both a humoral immune response, resulting in the production of antimicrobial peptides and other effector proteins, and a cellular immune response mediated by hemocytes and several other factors that result in phagocytosis or coagulative encapsulation, nodulation, and melanization [67–70]. Four major immune response pathways have been characterized in Dipteran species. The Toll and Imd signaling pathways lead to a humoral response activated primarily by Gram-positive bacteria and fungi or by Gram-negative bacteria, respectively. The JAK/STAT signaling pathway has a primary role in cellular immunity to a variety of microbes, and the RNA interference (RNAi) pathway is a major component of insect antiviral defense. Each of these pathways consists of recognition proteins that detect specific microbial components and initiate the appropriate intracellular signaling cascade that results in the production of specific immune effector molecules. Although the four pathways are distinct, they can interact in a coordinated fashion and intersect with other pathways during an immune response.

Major components of the *X. cheopis* immune system annotated in this study are listed in Supplementary File 7. The genome encodes the key elements of all four major immune signaling pathways. Sixteen pattern recognition protein genes were identified, including six classes of peptidoglycan recognition proteins (PGRP) and three classes of Gram-negative binding proteins (GNBP) predicted to interact with bacteria and fungi and initiate immune signaling pathways. Fourteen antimicrobial peptides (defensin, attacin, and coleoptericin classes) and eight lysozymes were annotated. All components of the IMD pathway were found. The protein MYD88, a component of the TOLL pathway was absent, but was found in the salivary and midgut transcriptomes of *X. cheopis* (Supplementary File 7). Regarding the JAK-STAT pathway, the proteins SOCS and PIAS were absent, but were found in the midgut transcriptome, as indicated in Supplementary File 7. The *Drosophila* protein unpaired, a component of the JAK-STAT pathway was also missing, but this protein appears to be specific to Brachycera flies, as shown by blasting the *Drosophila* sequence against all insect sequences present in the NCBI Non-redundant (NR) database (not shown). Genes involved in the generation of antibacterial reactive oxygen species (Duox-ROS system) were also identified. For the cell-mediated arm of insect immunity, several genes encoding hemocyte phagocytosis receptors and coagulation/melanization effectors (C-type lectins, four complement-like factors, including a thioester-containing protein (TEP), and prophenoloxidase pathway proteins) were also present (Supplementary File 6, Supplementary spread­sheet 1).

### Detoxification related genes

The detoxification of hormones and xenobiotics can be described in three phases: In Phase I the target compounds are converted to more hydrophilic products. In Phase II further conjugation of more hydrophilic units are added to the targets, which are excreted by cellular transporters in Phase III. Enzymes of Phase I detoxification include the multi gene families of cytochrome P450 (cyp450) and carboxylesterase (CaE), while in Phase II the Glutathione S-transferases (GST) are active [71]. Follows an account of these multi-gene families in both *X. cheopis* and *C. felis.*

Cytochrome P450 enzymes are heme-containing proteins that display monooxygenase activity [72], inflicting hydroxylation, epoxidation and dealkylation to their substrates including hormones, vitamins and foreign compounds. They contain the conserved motif F-x(2)-G-x(3)-C-x-G associated with the heme-binding domain, containing the cysteine that coordinates with the iron atom of the heme moiety. The reaction starts with a reduced iron in the heme, that loses an electron to the substate. This iron will be reduced from Fe^**3+**^ to Fe^**2+**^ by the enzyme NADPH-cytochrome P450 reductase, annotated in the *X. cheopis* genome as g81200.t1.

**Fig. 1:**
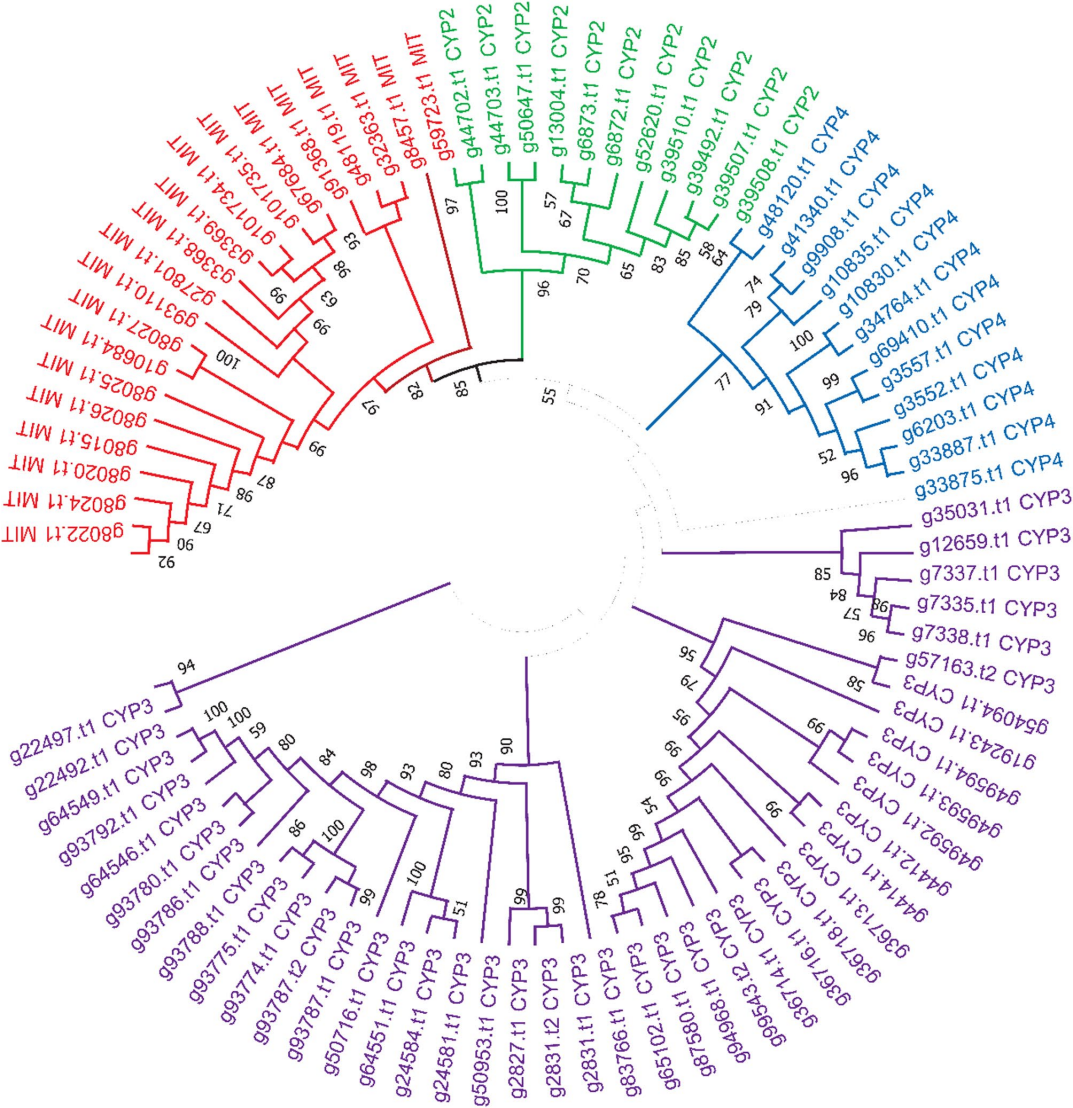
Evolutionary analysis of the *X. cheopis* cytochrome P 450 proteins. The evolutionary history was inferred by using the Maximum Likelihood method and JTT matrix-based model [75].The bootstrap consensus tree inferred from 1000 replicates is taken to represent the evolutionary history of the taxa analyzed [76]. This analysis involved 96 amino acid sequences. All positions with less than 50% site coverage were eliminated, i.e., fewer than 50% alignment gaps, missing data, and ambiguous bases were allowed at any position (partial deletion option). There were a total of 573 positions in the final dataset. Evolutionary analyses were conducted in MEGA X [34]

CypP450 are ancient enzymes, existing in bacteria and eukaryotes, their genomes expressing multiple copies, arranged in subfamilies or clades [73]. Four clades were identified in insects, the CYP3, CYP4, CYP2 and mitochondrial clades. Analysis of the *Drosophila melanogaster* genome revealed 90 genes coding for members of the Cyt P450 superfamily, 7 of which are probable pseudogenes [74]. The genome of *X. cheopis* has 85 genes that are members of the Cyt P450 superfamily, with 2 additional splice variants. According to their KOG matches, 54 of these gene products are in the CYP3 clade, 20 in the mitochondrial, 12 in the CYP4 and 11 in the CYP2 clade. We have also estimated the distribution of the Cyt P450 clades in the genome of *C. felis.* Comparison of these clade numbers with other insects indicate that *X. cheopis* has a full set of these enzymes. Phylogenetic analysis of the *X. cheopis* cyp450 protein products shows the four distinct clades clustering according to the KOG specification (Fig. 1).

Mitochondrial clade: Members of the mitochondrial clade usually act on hormones and vitamins [77–80]. The *X. cheopis* gene coding for the transcript g48119.t1 is similar to *D. melanogaster disembodied* which codes for a mitochondrial P450 transcript involved in ecdysteroid C_**22**_**-**hydroxylase activity. Similarly, g59723.t1 codes for a transcript best matching the *Drosophila shadow* gene, another mitochondrial CYPP450 enzyme with ecdysteroid C_**22**_-hydroxylase activity [81].

CYP2 clade: This clade also contains genes coding for transcripts displaying physiological functions. The gene coding for the g39492.t1 transcript is similar to the *D. melanogaster Phantom* gene, involved in the metabolism of insect hormones, for its ecdysteroid C_**25−**_hydroxylase activity [82]. It may be also capable of the breakdown of synthetic insecticides. The gene g50647.t1 codes for a CYP2 transcript similar to the *Drosophila spook* gene [83], which is involved in ecdysteroidogenesis.

CYP3 clade: This is the most numerous clade in insects, with many of its products metabolizing xenobiotics or insecticides.

Cyp4: This is also a numerous clade with many attributed functions in *Drosophila*, as metabolizers of xenobiotics, odorants, and pheromones.

The carboxylesterases (CaEs) are hydrolytic enzymes with the α/β hydrolase fold, sharing a 2-step serine hydrolase mechanism and active site consisting of a Ser, His and a negatively charged Asp or Glu residue. This family include cholinesterases and lipases, among a diversity of other enzymes. There are also members of this family that lack enzymatic activity, as with some cholinesterases that sequester organophosphate insecticides [84]. The genome of *X. cheopis* has 78 transcripts matching members of the ESTHER database [25]. The classes Carb_B_arthropoda (24 genes), Neuroligin (15 genes) and Acidic_Lipase (9 genes) were the most abundant (Supplementary File 6, Supplementary spread­sheet 1). We highlight the finding of the gene coding for the enzyme PAF acetyl hydrolase which was found in both *X. cheopis* and *C. felis*, and in the louse *Pediculus humanus*, but not in mosquitoes, sand flies, tsetse, ticks, or blood feeding bugs. Platelet activating factor (PAF) is produced by activated platelets and leukocytes, having pro-inflammatory activity [85, 86]. Its activity in the salivary glands of *C. felis* has been previously described [87].

Glutathione S-transferases are enzymes involved in the detoxification of endogenous and xenobiotic compounds [88]. They act through several mechanisms, the most common being the glutathione conjugation reaction, where they catalyze the nucleophilic attack of the thiol group of reduced glutathione to the electrophilic center of chemical substrate, thus removing the charged center of the substrate and affecting its solvent solubility. GST-based detoxification may also occur through the glutathione is used as a cofactor to remove a hydrogen atom from the substrate leading to the elimination of chlorine. Some GSTs can also function as a peroxidase, thus avoiding oxidant stress. Finally, some GSTs are found that do not have any enzymatic activity but neutralize their targets by binding to them with high affinity, a process akin to kratagonists [89]. Two domains are identified in GSTs: a N-terminal thioredoxin-like domain and a C-terminal alpha-helical domain associated with substrate binding. These enzymes are expressed mainly in the cytoplasm, but mitochondrial, nuclear and dehydrochlorination reaction, occurring in the detoxification of organic chlorine insecticides, where reduced microsomal forms have been described [90]. In *Drosophila*, only one member of this family is microsomal, while 37 were identified as cytosolic. The flea *C. felis* has 21 putative cytosolic GSTs, while 28 were found in the mosquito *A. gambiae* and 11 in *Apis mellifera*. The genome of *X. cheopis* revealed 17 genes coding for cytoplasmic glutathione S transferases, identified by having both amino and carboxyterminal prosite motifs for soluble glutathione S transferase enzymes [91].

**Fig. 2:**
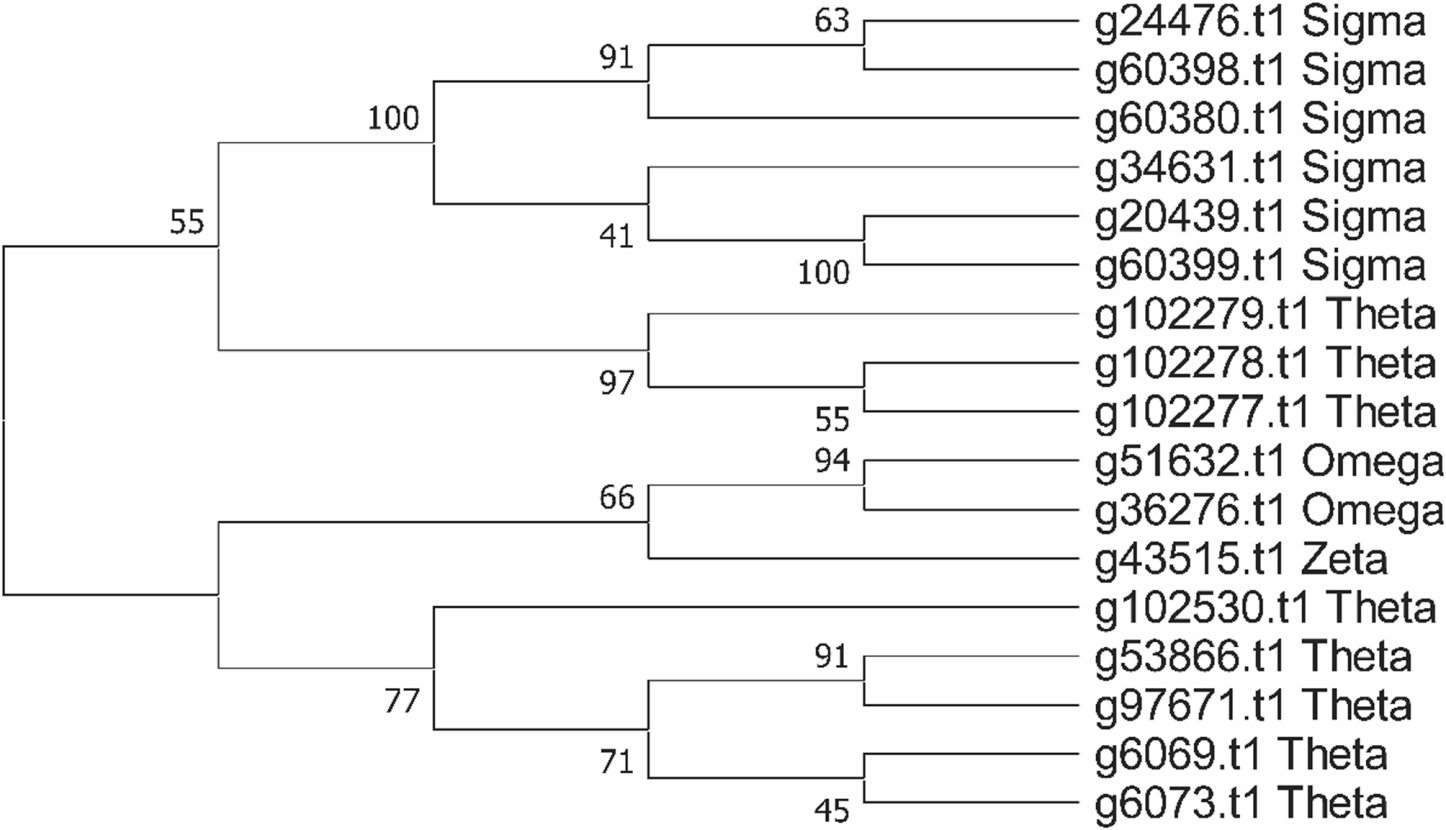
Evolutionary analysis of the *X. cheopis* Glutathione S transferase proteins. The evolutionary history was inferred using the Neighbor-Joining method [76]. The bootstrap consensus tree inferred from 1000 replicates [76] is taken to represent the evolutionary history of the taxa analyzed [76]. This analysis involved 38 amino acid sequences. All ambiguous positions were removed for each sequence pair (pairwise deletion option). There were a total of 650 positions in the final dataset. Evolutionary analyses were conducted in MEGA X [34]

In insects, this gene family is divided into six subclasses: Delta, Epsilon, Sigma, Omega, Theta, and Zeta [71]. In fleas, the Sigma subclass is the most abundant with 6 genes in *X. cheopis* and 7 in *C. felis*, followed by the Delta subclass (5 genes in *X.c.* and 5 genes in *C.f*,), the Omega (1 gene in *X.c.* and 3 genes in *C.f.*) and the Zeta subclass with one gene in *X.c.* and 2 genes in *C.f.* No member of the Epsilon class was found. Alignment and phylogenetic analysis of the flea GSTs shows that the Sigma class is in over two branches, indicating a possible additional subclass in fleas (Fig. 2).

## Discussion

We present here a draft genome of *X. cheopis*, in which 95,638 putative CDS were identified and functionally classified. Notably, the number of predicted CDS is significantly higher than that reported for the *C. felis* genome [4]. One contributing factor may be the larger estimated genome size of *X. cheopis*, which ranges from 800 to 900 Mb, compared to the estimated 430–550 Mb of the *C. felis* genome [4]. In addition, our analysis revealed a high number of predicted CDS with substantial sequence similarity. Specifically, 15,886 CDS share at least 95% sequence identity, and 28,252 share at least 90%, which contributes to the inflated number of putative CDS in the current assembly.

In addition to *X. cheopis* genome, we also identified sequences belonging to the rat flea symbiont. Phylogenetic analysis of the symbiont of *X. cheopis* indicate it is a divergent member of the *Wolbachia* genus, as are the symbionts of the cat flea *Ctenocephalides felis*, the springtail *Folsomia candida*, the banana aphid *Pentalonia nigronervosa* and the nematode from the genus *Howardula*. With additional disclosures of novel *Wolbachia* genomes, these “solitary” species may “find” their trees, and shed light on the evolution of this peculiar genus.

Obligate blood-feeders, such as the *X. cheopis* adults analyzed in this study, rely on a nutritional unbalanced diet to fulfill their metabolic needs. To overcome this deficiency and complement their diet with essential nutrients, hematophagous arthropods have evolved in association with nutritional endosymbionts, which may provide their hosts with B vitamins and cofactors [92]. While some endosymbionts are essential for host development and survival, others are facultative, enhancing host reproductive fitness [36, 93, 94].

The genome of the *X. cheopis Wolbachia* endosymbiont indicates it retains the complete biosynthesis operon for riboflavin, suggesting it may act as a nutritional mutualist. Riboflavin is a precursor of the cofactors FAD and FMN and it is involved in the metabolism of carbohydrates, proteins, and lipids. In *Cimex lectularius* it has been shown that riboflavin provided by the *Wolbachia* endosymbiont is required for the insect growth, survival and reproduction [95]. While riboflavin genes are highly conserved in insect-associated *Wolbachia* genomes, those for the biosynthesis of other B vitamins are usually rare and presumably originated via LGT (lateral gene transfer) [95]. Like the majority of *Wolbachia* strains, the genome of *X. cheopis Wolbachia* showed to be missing genes involved in the production of most B vitamins indicating that these biosynthetic pathways may be non-functional [95]. The limited metabolic capacity of *X. cheopis Wolbachia* might suggest that other members of the flea microbiome could be providing the host with vitamins deficient in the blood. The potential role of *Wolbachia* as a nutritional symbiont in *X. cheopis* as well as the interaction between the rat flea and other members of the indigenous microbial community remains to be investigated.

The analysis of TE abundance in the *C. felis* genome, using the same tools used for the *X. cheopis* genome, revealed decreased TE abundance when compared to the rat flea genome where we found 4% of the GC by Class I elements and 4% of the GC by Class II elements. The frequency distribution of the families, however, are similar to those found in the *X. cheopis* genome. These differences are not surprising since even among closely related *Drosophila* species, the GC ranges from 40% (in *D. ananassae*) to 10% (in *D. miranda* and *D. simulans*) [96].

Blood acquisition is a complex process for hematophagous organisms, requiring them to overcome the host’s immune and hemostatic responses to obtain a blood meal. Functional characterization of salivary proteins from blood-feeding arthropods revealed that such protein often possess potent pharmacological activity, capable of disrupting vasoconstriction, platelet activation, and blood clotting, thereby facilitating successful blood feeding [97]. In fleas, acid phosphatases and members of the FS-H/I families have been shown to be among the most prevalent proteins within the salivary glands [49, 98, 99]. Acid phosphatase activity have been reported in the salivary gland homogenates of other hematophagous vectors, such as ticks and triatomines; however, their contribution to blood acquisition remains unclear [100, 101]. Functional characterization of *X. cheopis* acid phosphatases has shown that they can bind to biogenic amines and leukotrienes, thereby limiting their availability [102] and interfering with host’s homeostatic and immune responses. Similarly, FS-H/I appears to act as channel blockers [103, 104], potentially modulating the host nociception and immune responses [105, 106].

The abundance of these two protein families in flea salivary glands, along their tandem organization within the same supercontig, suggests that these genes occupy genomic regions prone to rapid evolution– likely driven by gene duplication events– which has led to their expansion. Similar patterns of gene duplication and expansion have also been proposed for salivary glands in other blood-feeding arthropods [107].

Blood meal digestion in fleas remains relatively understudied compared to other blood-feeding arthropods such as mosquitoes [108, 109], kissing bugs [110] and ticks [111, 112]. The current understanding of rat flea blood digestion is based on histological changes observed in the flea midgut and food bolus at various time points after feeding and is subdivided into three main stages [113]. The initial stage, encompasses the period from the end of blood intake until lysis of red blood cells, typically lasting around 3 h. During this phase, the flea midgut expands, becomes visibly distended, and eventually becomes fully engorged with blood [55]. During stage II, the contents of the midgut are processed by digestion, transforming the bright red blood to a darker brown. Finally, stage III is marked by the presence of remnants from the digested blood and formation of hematin, in some cases the midgut is devoid of content. One limitation is that these observations are based on *X. cheopis* that consumed a single host (mouse) blood meal and variability in the duration of the phases is observed following feeding on the blood of other rodents. This variability is likely dependent on the lipid composition of the red blood cells and the flea’s degree of adaptation to a particular host [114, 115]. Our sequencing of the rat flea genome will allow researchers to expand upon observational descriptions of flea bloodmeal digestion and to track and match the temporal expression of physiologically relevant digestive enzyme, peritrophin, and heme sequestration genes to each stage. The main host of *X. cheopis* are rats (*Rattus spp.*), which, along with the *Sciuridae*, tend to have poorly soluble hemoglobin molecules [116]. Hemoglobin of the brown rat (*Rattus norvegicus*) will rapidly form rhomboid-shaped crystals following red blood cell lysis *in vitro.* These oxyhemoglobin crystals can be observed in the gut of rodent fleas shortly after the ingestion and lysis of brown rat RBCs [117]. The crystals are eventually solubilized in the flea midgut; however, this intrinsic property of brown rat hemoglobin may moderate the initial burst of heme-derived ROS produced during blood digestion, and may delay expression, diminish transcription levels, and/or limit the requirement for genes with antioxidant function.

The function of flea peritrophin genes expressed following blood-feeding remains cryptic. If the *X. cheopis* peritrophins and mucin-like proteins are not involved in PM formation, further investigation is needed to determine what role, if any, these proteins play in blood meal digestion. We have hypothesized previously that some of the rat flea peritrophins may be important for stability of the chitinous tracheal cuticle and moderating respiration during infection with *Y. pestis* [56].

The *X. cheopis* genome appears to encode the major immune response pathways that have been established for other insects (Imd, Toll, Duox/ROS, JAK/STAT, RNAi, and Cell-mediated/phagocytosis; Supplementary File 6, Supplementary spread­sheet 1). To date, few studies have directly examined the immune response of fleas to the bacterial pathogens and other microbes that they encounter. Infection of *X. cheopis* with *Y. pestis* in infected blood meals induces the Imd immune signaling pathway resulting in the production of antimicrobial peptides and the Duox/ROS system [56, 118]. Direct injection of *Y. pestis* or *E. coli* into the *X. cheopis* hemocoel also results in an antibacterial peptide response [118, 119]. Similar challenges of the cat flea *C. felis* with a variety of Gram-negative and Gram-positive bacteria have documented the upregulation of genes in the Imd, Toll, Duox/ROS, and Cell-mediated/phagocytosis pathways, suggesting that this flea too has the full armamentarium of insect immune responses [120–123].

The annotation of the *X. cheopis* genome for detoxification enzymes associated with metabolic and/or insecticide detoxification revealed a full set of enzymes associated with multi gene families of cytochrome P450 (cyp450), carboxylesterase (CaE) and Glutathione S-transferases (GST). This database should be of interest for future studies on rat flea insecticide resistance mechanisms.

In summary, we identified several notable genetic features of the rat flea that provide useful insight and starting points for new investigations into *X. cheopis* vector biology. These observations include: (1) Higher proportions of transposable elements in rat fleas than those observed in cat fleas. (2) Abundant salivary protein families identified in tandem in the same supercontig; suggestive of rapid evolution potentially driven by gene duplication and tandem recombination. (3) The presence of multiple peritrophin genes with chitin-binding domains, despite fleas not producing a peritrophic matrix, suggest a cryptic yet important function in flea physiology. (4) The serine peptidase profile of rat fleas indicates they are the digestive enzymes of primary importance, however, some of these lack catalytic residues and are likely enriched in the midgut. (5) Rat fleas have all the genes needed to synthesize heme, however, the presence of a novel putative heme binding protein unique to *X. cheopis* potentially indicates some ability to sequester or monitor heme concentrations, unlike the cat flea which may avoid this potential requirement by rapidly digesting and defecating blood. (7) *X. cheopis* appear capable of producing all canonical insect immune responses to invading microorganisms. (8) Identification of a platelet activating factor (PAF) acetyl hydrolase unique to rat fleas, cat fleas, and human head and body lice, but not other blood-feeding arthropods suggests a potentially important role for this protein in blood acquisition for these insects. Sequencing and disclosure of the *X. cheopis* genome will be valuable in advancing research on this important vector insect and the Siphonaptera more generally.

## Supplementary Information

Supplementary File 1: Supplementary Figure 1: Phylogram obtained from the coding sequences from members of the *Wolbachia* endosymbionts rooted on *Anaplasma*, using the GToTree phylogenomic method. The numbers on the branches represent the bootstrap support. The *Wolbachia *symbionts of fleas are marked with a red square symbol. See supplementary file 1 for NCBI accessions of the genomes.

Supplementary File 2: List of Rickettsiales organisms used for genomic comparisons of Supplementary figure 1.

Supplementary File 3: List of orthologous proteins between *Xenopsylla cheopis *and *Ctenocephalides felis.*

Supplementary File 4: Transposable elements from* Xenopsylla cheopis *identified by Repeat Masker.

Supplementary File 5: Peritrophins/chitin-binding from *Xenopsylla cheopis.*

Supplementary File 6: Supplementary Spreadsheet 1. Hyperlinked spreadsheet with predicted proteins and coding sequences of *Xenopsylla cheopis.*

Supplementary File 7: Immunity related genes of *Xenopsylla cheopis.*

## Data Availability

The Xenopsylla cheopis Whole Genome Shotgun project has been deposited at DDBJ/ENA/GenBankunder the accession JBGSYW000000000, Bioproject PRJNA1105622 and Biosample SAMN41953765. The version described in this paper is version JBGSYW010000000. The genome for the Wolbachia endosymbiont of Xenopsylla cheopis has been deposited at DDBJ/ENA/GenBank under the accession CP168948, Bioproject PRJNA1050211 and Biosample SAMN38728102.
