# Supplementary material for: An insight into the draft genome of the Oriental rat flea, Xenopsylla cheopis, together with its Wolbachia endosymbiont
Source: BMC Genomics. 2025 Jul 1;26:621. doi: 10.1186/s12864-025-11759-8 (PMC12211885; doi:10.1186/s12864-025-11759-8)
Supplement: Supplementary file 5 — Peritrophins/chitin-binding from Xenopsylla cheopis. [file 12864_2025_11759_MOESM5_ESM.docx]

**Supplementary file 5:** Peritrophins/chitin-binding from *Xenopsylla cheopis*.

*Determined from RNA-seq data collected from reference [1]. Genes listed in descending order of most to least abundant transcript.

| **CDS Name** | **Encoded Protein** | **Detected in rat flea midgut transcriptome?**  **(≥5 Avg TPM)*** | **E Value** | **Coverage (%)** | **Protein Database** |
| --- | --- | --- | --- | --- | --- |
| g53936.t1 | Peritrophin-1 | Y | 5.00E-11 | 98.8 | DIPTERA |
| g59669.t1 | Peritrophin-48-like | Y | 0 | 94.9 | FLEAS |
| g97975.t1 | Peritrophin-1 | Y | 9.00E-16 | 83 | UNIPROTKB |
| g88299.t1 | Peritrophin-like protein 3 | Y | 2.00E-77 | 100 | FLEAS |
| g59660.t1 | Peritrophin-48-like | Y | 3.00E-65 | 39.4 | FLEAS |
| g59663.t1 | Peritrophin-48-like | Y | 0 | 65.5 | FLEAS |
| g62481.t1 | Peritrophin-48 partial | Y | 9.00E-30 | 110.1 | DIPTERA |
| g7878.t1 | Peritrophin-1 | N | 0 | 78.2 | DIPTERA |
| g68954.t1 | Peritrophin-1 partial | N | 3.00E-67 | 63.8 | DIPTERA |
| g53923.t1 | Peritrophin-like protein 3 | N | 3.00E-24 | 72 | FLEAS |
| g91023.t1 | Peritrophin-1 | N | 2.00E-15 | 93.4 | UNIPROTKB |
| g55284.t1 | Peritrophin-1-like | N | 3.00E-87 | 68.4 | DIPTERA |
| g43826.t1 | Peritrophin-48-like | N | 3.00E-65 | 100.8 | DIPTERA |
| g47884.t2 | Peritrophin-like protein | N | 1.00E-38 | 82.8 | DIPTERA |

1. Bland DM, Martens CA, Virtaneva K, Kanakabandi K, Long D, Rosenke R, Saturday GA, Hoyt FH, Bruno DP, Ribeiro JM *et al*: **Transcriptomic profiling of the digestive tract of the rat flea, *Xenopsylla cheopis*, following blood feeding and infection with *Yersinia pestis***. *PLoS neglected tropical diseases* 2020, **14**(9):e0008688.
